# A Systematic Review of Plants Used for the Treatment of Diarrhea in Mozambique

**DOI:** 10.1155/bmri/4132094

**Published:** 2026-03-08

**Authors:** Adilência Mataveia, Filomena Barbosa, Sílvia Langa, Custódio Bila, Valeriano Chichava, Natália Ngome, Mércia Inroga, Helena Correia, Delfina Hlashwayo

**Affiliations:** ^1^ Departamento de Ciências Biológicas, Faculdade de Ciências, Universidade Eduardo Mondlane, Maputo, Mozambique, uem.mz; ^2^ Faculdade de Veterinária, Universidade Eduardo Mondlane, Maputo, Mozambique, uem.mz; ^3^ Direcção de Pesquisa em Saúde e Bem-Estar, Instituto Nacional de Saúde, Maputo, MISAU, Maputo, Mozambique; ^4^ Centro de Investigação e Desenvolvimento em Etnobotânica, Namaacha, Mozambique

**Keywords:** diarrhea, ethnobotany, ethnopharmacology, medicinal plants, Mozambique, traditional medicine

## Abstract

**Introduction:**

Diarrhea is one of the leading causes of mortality, particularly among children under 5 years of age in developing countries. The increasing prevalence of antimicrobial resistance has complicated the search for effective treatments, highlighting medicinal plants as a promising source for novel therapeutic agents.

**Aim:**

This systematic review is aimed at identifying plant species traditionally used to treat diarrhea in Mozambique and at documenting their ethnomedicinal characteristics.

**Methodology:**

A comprehensive literature search was conducted to gather information on traditional plants used for diarrhea in Mozambique. Relevant publications up to 10 April 2024 were retrieved from PubMed, ScienceDirect, libraries, and online repositories. The review was performed in accordance with the PRISMA guidelines.

**Results:**

In this study, a total of 174 plants were identified as being used for the treatment of diarrhea in Mozambique, including 12 identified only to the genus level, one variety, and three subspecies. An additional 10 species were reported solely by their vernacular names. *Terminalia sericea* was the plant most frequently cited species, appearing in 14 of the 38 studies. Roots were the commonly used plant part, accounting for 49.8% of reported uses, with decoction being the predominant method of preparation (45.8%). Oral administration was the most common route, although other methods were reported. Some species identified in this review are at risk of extinction, emphasizing the need for conservation efforts.

**Conclusions:**

The study highlights the diverse range of plant traditionally used to manage diarrhea in Mozambique. These species represent promising candidates for future pharmacological and clinical research, offering potential solutions not only within Mozambique but also for addressing diarrheal diseases on a broader, global scale.

## 1. Introduction

Diarrhea, defined as the passage of three or more loose or liquid stools per day or more frequently than normal for an individual [1], remains a leading cause of mortality worldwide, particularly among children under 5 years of age. In 2019, diarrhea was the second leading cause of death in this age group, resulting in approximately 370,000 deaths globally [2].

In Mozambique, diarrhea represents approximately 20% of hospital admissions as is associated with substantial morbidity and mortality, especially in children under five [3, 4]. Recent studies have also highlighted the burden of diarrhea in adults, emphasizing its impact on public health in the country [5, 6].

Diarrhea has diverse etiologies, including infections agents, dietary imbalances, food intolerances, and gastrointestinal disorders. Acute diarrhea is most often infectious in origin [7]. Bacterial pathogens such as *Salmonella* spp., *Shigella* spp., *Escherichia coli*, *Campylobacter* spp., and *Vibrio cholerae* are well‐established causes, whereas protozoa including *Giardia* spp., *Cryptosporidium* spp., and *Cyclospora* spp. also contribute to diarrheal illnesses. Viral agents such as rotavirus, astrovirus, norovirus, and adenovirus are recognized as common causes of viral gastroenteritis and associated diarrheal episodes [3, 8]. In contrast, chronic diarrhea, persisting for more than 2 weeks, is noninfectious and may result from nutrient malabsorption, chronic inflammatory conditions (e.g., inflammatory bowel disease), or adverse drug effects [7].

Treatment of diarrhea primarily involves fluid and electrolyte replacement [8]. In some cases of acute and persistent diarrhea caused by bacterial and protozoal infections, antibiotics may be indicated [7]. However, the global rise in antimicrobial resistance among diarrhea‐causing microorganisms poses a serious challenge, including in sub‐Saharan Africa [6, 9–11].

In Mozambique, enteropathogenic microorganisms increasingly exhibit multidrug resistance, including to antibiotics used to treat infectious diarrhea [6, 12–15]. This trend complicates disease management and highlights the urgent need for effective alternative strategies. The World Health Organization (WHO) has identified several enteropathogenic bacteria associated with diarrhea as priority pathogens for the development of novel antibiotics [16].

Bioactive compounds from plants may offer alternative antibacterial, antiprotozoal, and gastrointestinal‐regulating properties, providing a promising avenue to address antimicrobial resistance and improve diarrhea management [17, 18].

Traditional medicine is widely used across Africa due to its accessibility, affordability, and cultural acceptance [19, 20]. Medicinal plants remain central to healthcare, with over 60% of the Mozambican population relying on traditional medicine for primary healthcare needs [21]. Mozambique hosts a rich botanical diversity, comprising 7,099 vascular plant taxa, of which 731 are employed for medicinal purposes [22, 23].

Identifying the specific plants traditionally used to treat diarrhea in Mozambique aligns with the WHO′s efforts to strengthen the evidence base for traditional, complementary, and integrative medicine [24] and supports the discovery of new bioactive compounds with potential antidiarrheal, antimicrobial, anti‐inflammatory, and gastrointestinal‐regulating properties, ultimately informing evidence‐based interventions for diarrheal diseases.

The primary objective of this systematic review was to identify and document plants species used for the treatment of diarrhea in Mozambique, including ethnomedicinal information, with the aim of providing a comprehensive resource for future pharmacological research and evidence‐based applications.

## 2. Methods

### 2.1. Data Collection

A systematic review was conducted to compile information on medicinal plants used to treat diarrhea in Mozambique. Multiple sources were consulted, including books, scientific articles, undergraduate theses, postgraduate theses, and scientific reports, covering publications up to April 10, 2024 without language restrictions.

The review followed the Preferred Reporting Items for Systematic Reviews and Meta‐Analyses (PRISMA) guidelines [25]. The PRISMA checklist is available in Supporting Information 1.

To identify relevant literature, institutional libraries were consulted, including the Mozambican Agricultural Research Institute (*Instituto de Investigação Agrária de Moçambique-IIAM*), the LMU Herbarium Library at Eduardo Mondlane University, and the National Directorate of Traditional and Alternative Medicine. Additional theses and dissertations were assessed via online repositories: *Repositório Saber* (http://www.saber.ac.mz), *Repositório Científico da Universidade Católica de Moçambique* (http://repositorio.ucm.ac.mz), and *Repositório da UEM* (http://www.repositorio.uem.mz).

Scientific articles were retrieved from PubMed and ScienceDirect. The detailed search strategy is provided in Supporting Information 2. Screening of studies was independently conducted by two authors (A.M. and D.H.).

All studies reporting plant use for diarrhea, regardless of etiology, were included. Information was extracted and compiled into a database, including capturing plant parts used, modes of preparation, and routes of administration. Studies were excluded if they were review articles or lacked relevant information on plant use. Only plants identified as main species were included in the quantitative analyses; plants mentioned solely as secondary components of preparation methods are reported in Supporting Information 3, which constitutes the complete study database.

Scientific names were verified using the World Flora Online Plant List (https://wfoplantlist.org/plant-list/) as of 19 April, 2024, and botanical families followed the APG IV system (Angiosperm Phylogeny Group 2016) [26]. Conservation status was checked on April 20, 2024 via the IUCN Red List (https://www.iucnredlist.org), and species origin and endemism were verified using the updated checklist of Mozambique′s vascular plants [22].

### 2.2. Data Storage and Analysis

Data were systematically organized using Microsoft Word 2021 and subsequently analyzed with Microsoft Excel 2021.

### 2.3. Percentage of Citations

To identify the most frequently cited plants, the percentage of citations was calculated as follows:

Percentage of citation = (number of studies citing the plant/total number of studies) ∗ 100.

In this review, plants cited in more than 15% of studies were considered frequently cited.

### 2.4. Commonly Used Plant Parts

The relative use of different plant parts was calculated as follows:

Percentage of plant parts used = (number of citations for a specific plant part/total citations of all plant parts) ∗ 100.

Each plant part cited was counted individually, and the denominator represents the cumulative count of all plant parts reported.

### 2.5. Common Methods of Preparation

The percentage of preparation methods was calculated as follows:

Percentage of preparation mode = (number of citations for a specific preparation/total citations of all methods) ∗ 100.

Each citation of a preparation method was counted individually. The total number of citations represents the cumulative count of all modes of preparation used.

### 2.6. Geographic Distribution Mapping

To visualize the geographic distribution of medicinal plant citation records in Mozambique, we gathered information on species occurrences as described in the included studies. The coordinates obtained representing these locations were plotted in Google Earth and saved in the KML extension. Subsequently, the coordinate data in KML format were exported to the ArcGIS 10.4 program, facilitating the conversion from KML to Shapefile format for further analysis. To enhance precision, specific Mozambique vector files were selected. The geographic coordinates were then overlaid onto these vector files. To visualize and communicate the distribution effectively, the map composer feature was activated in ArcGIS, resulting in the creation of a comprehensive geographic map demonstrating the plant citation records.

## 3. Results

### 3.1. Identified Studies

**Figure 1:**
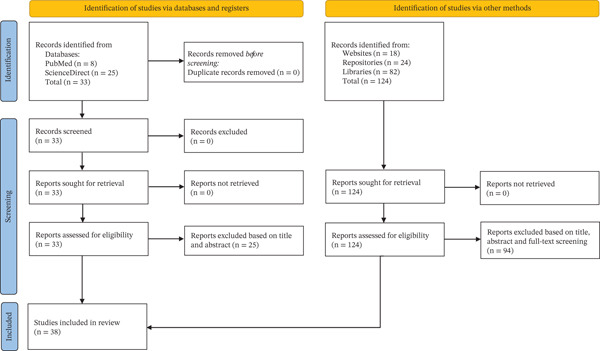
PRISMA flow diagram.

A total of 157 studies were initially retrieved through database searches and other sources. After applying the inclusion criteria, 38 studies [27–64] were retained for this systematic review. The remaining 119 studies were excluded for the following reasons: 90 were removed at the title screening stage (37 conducted outside Mozambique, 52 not addressing medicinal plants, and 1 review article), 23 were excluded after abstract review (11 did not include medicinal plants, 10 lacked information on plant use, and 2 were review studies), and 6 were excluded following full text assessment (4 did not specify the type of disease treated and 2 did not include plants used for diarrhea). The study selection process is illustrated in the PRISMA flow diagram (Figure 1).

Most included studies were undergraduate theses (*n* = 18) [27, 36, 37, 43, 44, 46, 48, 49, 51, 53–57, 59, 61–63], followed by scientific articles (*n* = 13) [28, 34, 35, 38–41, 45, 47, 52, 58, 60, 64], five books [29–33] on medicinal plants and their traditional use in Mozambique, and two technical reports [42, 50]. Undergraduate theses were included as they provide valuable ethnobotanical data that remain largely undocumented in Mozambique′s published literature.

Temporal trends in reporting revealed fluctuating patterns. Before 1990, only three records were identified [29, 30, 52]. Between 1990 and 1999, nine records were documented [27, 31, 32, 46, 49, 54, 55, 57, 61]. The period 2000–2009 showed a notable increase, with 14 records [33, 34, 36, 37, 39, 42–44, 48, 51, 56, 59, 62, 63]. From 2010 to 2019, seven records were documented [28, 38, 41, 47, 53, 58, 64] and between 2020 and 2022 four records were identified [35, 40, 45, 60]. One report did not present the publication year [50].

### 3.2. Plants Used to Treat Diarrhea in Mozambique

A total of 174 main plants were identified as being used for the treatment of diarrhea in Mozambique, including 12 plants identified only to the genus level, one variety, and three subspecies. These plants belong to 59 different botanical families. An additional 10 plants could not be identified beyond their vernacular names and were therefore excluded from the quantitative analysis presented in this manuscript.

A comprehensive list of all identified species is provided in Supporting Information 3, which includes scientific and vernacular names, botanical families, vernacular names, plant parts used, preparation methods, IUCN conservation status, origin, endemism, occurrences records, percentage of citations, and references.

Regarding citation percentage, 105 species were cited in only one study, 31 were cited twice, 17 were cited three times, 5 were cited four times, and another 5 were cited five times. Notably, 11 species were cited in six or more studies, corresponding to a citation percentage ≥ 15.8%, and are considered frequently cited. These frequently cited species are listed in Table 1.

**Table 1:** Most cited plants used for the treatment of diarrhea.

Scientific name	Vernacular names (language)^a^	Botanical family	Parts used	Preparation methods	IUCN status	Native or introduced	No. of reports citing (%)
*Terminalia sericea* Burch. ex DC.	*Conola*	Combretaceae	Roots and leaves	Decoction^b^, infusion, and maceration, corn porridge prepared with maceration	Least concern	Native	14 (36.8%)
*Annona senegalensis* Pers.	Ata silvestre (port), *rompfa*	Annonaceae	Roots, (including root bark), stem, and leaves	Decoction, maceration, charring, and subsequent addition to food, corn porridge prepared with maceration	Least concern	Native	12 (31.6%)
*Vernonia colorata* Drake	*Nhatelo, pahlakufa*	Asteraceae	Roots and leaves	Decoction and maceration^c^	Least concern	Native^d^	10 (26.3%)
*Ozoroa obovata* (Oliv.) R.Fern. & A.Fern.	*Chinungumafi*	Anacardiaceae	Roots, leaves, and stem bark	Decoction and maceration^e^	Least concern	Native	9 (23.7%)
*Sclerocarya birrea* Hochst.	*Ncanhu*	Anacardiaceae	Leaves, roots, and bark	Decoction^f^	Not reported	Native	9 (23.7%)
*Garcinia livingstonei* T.Anderson	*Mbimbe*	Clusiaceae	Roots, stem, and bark	Decoction	Least concern	Native	8 (21.1%)
*Combretum molle* R.Br. ex G.Don	*Xicucutsi*	Combretaceae	Roots	Decoction and corn porridge prepared with decoction	Least concern	Native	7 (18.4%)
*Gymnosporia senegalensis* Loes.	*Xilhangua*	Celastraceae	Roots and leaves	Infusion	Least concern	Native	7 (18.4%)
*Tabernaemontana elegans* Stapf	*Nkahlo*	Apocynaceae	Roots	Decoction, infusion, and maceration;	Least concern	Native	7 (18.4%)
*Catharanthus roseus* (L.) G.Don	Beijo‐de‐mulata (port)	Apocynaceae	Whole plant and root	Decoction	Not reported	Introduced	6 (15.8%)
*Psidium guajava* L.	Goiaba (port)	Myrtaceae	Roots, fruit, stem, and leaves	Decoction	Least concern	Introduced	6 (15.8%)

^a^Vernacular names in italic correspond to terms reported in *Xichangana*, a native language from southern Mozambique. Vernacular names shown in parentheses (port.) indicate Portuguese. These species have additional vernacular names beyond those listed in Table 1 (please refer to Supporting Information 3). Further linguistic and regional details can be found in the original reports.

^b^The decoction can also be used as enema [27]; other plants can be added to decoction [27, 48, 53] including the root of *Tabernaemontana elegans* [32].

^c^In addition to ingestion, maceration can also be applied to the anus. Mixtures may be added [27, 53].

^d^Selected varieties or subspecies are native.

^e^Can be mixed with other plants [27, 46].

^f^Can include *Garcinia livingstonei* bark [46], scrape before decoction.

*Terminalia sericea* emerged as the most frequently cited species, reported in 14 of the 38 studies [27, 28, 32, 34–38, 42, 46, 48, 53, 55, 59]. This native Mozambican species is widely recognized and traditionally used, highlighting its importance in diarrhea management in the country. Most other frequently cited species are also native, with the exception of two introduced species: *Catharanthus roseus*, native to Madagascar and cultivated for both ornamental and medicinal purposes and *Psidium guajava*, originating from the Americas, whose fruit is widely consumed globally.

Fabaceae was the most represented botanical family, comprising 19 species. This was followed by Combretaceae with 13 species, and Anacardiaceae and Apocynaceae, each represented by 12 species.

### 3.3. Plant Parts Used, Preparation Methods, and Routes of Administration

**Figure 2:**
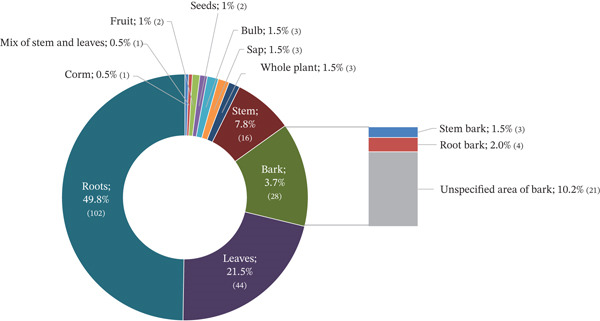
Percentage distribution of plant parts used for the treatment of diarrhea in Mozambique.

Roots were the most frequently used plant part, accounting for 49.8% of reported uses, followed by leaves (21.5%) and bark (13.7%). The relative distribution of plant parts used for diarrhea treatment in Mozambique is shown in Figure 2.

**Figure 3:**
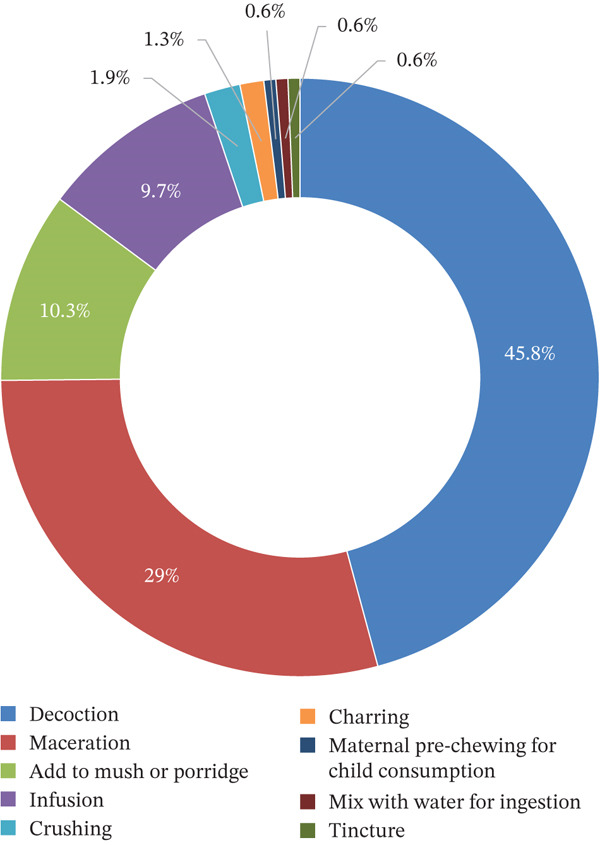
Overview of preparation methods for plants used in the treatment of diarrhea in Mozambique.

A range of preparation methods was reported across the included studies. Decoction was the predominant preparation method, representing 45.8% of preparations, followed by maceration (29%). The full range of preparation methods and their proportions are presented in Figure 3. Notably, some studies described distinct practices, such as maternal prechewing of plant material for administration to children, which may raise concerns regarding the potential transmission of infectious agents.

**Figure 4:**
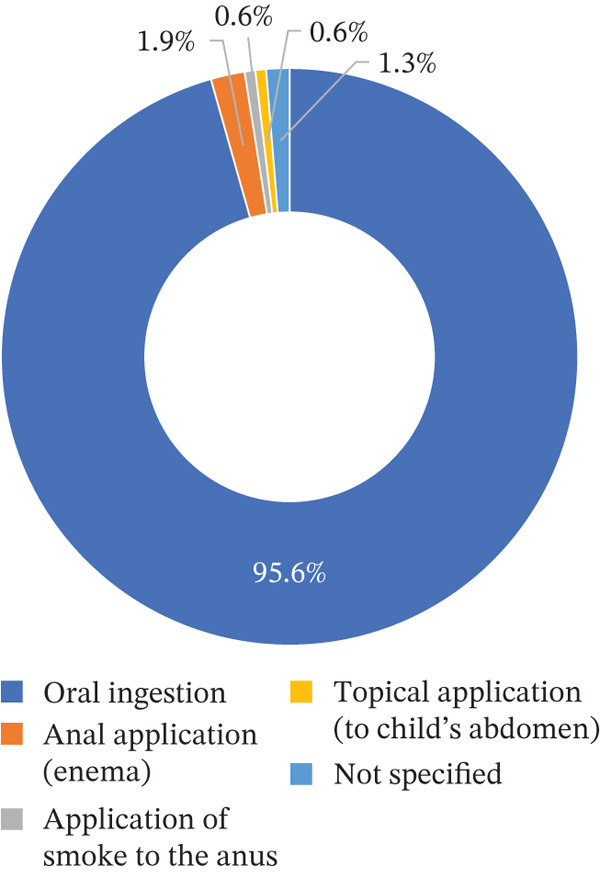
Overview of administration routes for plants used in the treatment of diarrhea in Mozambique.

With regard to routes of administration, decoctions and infusions assumed to be administered orally unless otherwise specified. Overall, oral ingestion accounted for 95.6% of reported administration. Less common routes included enema use in three cases (2.5%), one report of topical application of a prechewed preparation to a child′s abdomen, and one report describing the application of smoke to the anus (Figure 4). The latter involved burning the stem of *Spirostachys africana* with chicken feces on charcoal and directing the smoke to the anal region [27].

### 3.4. Geographical Distribution, Origin and IUCN Conservation Status

**Figure 5:**
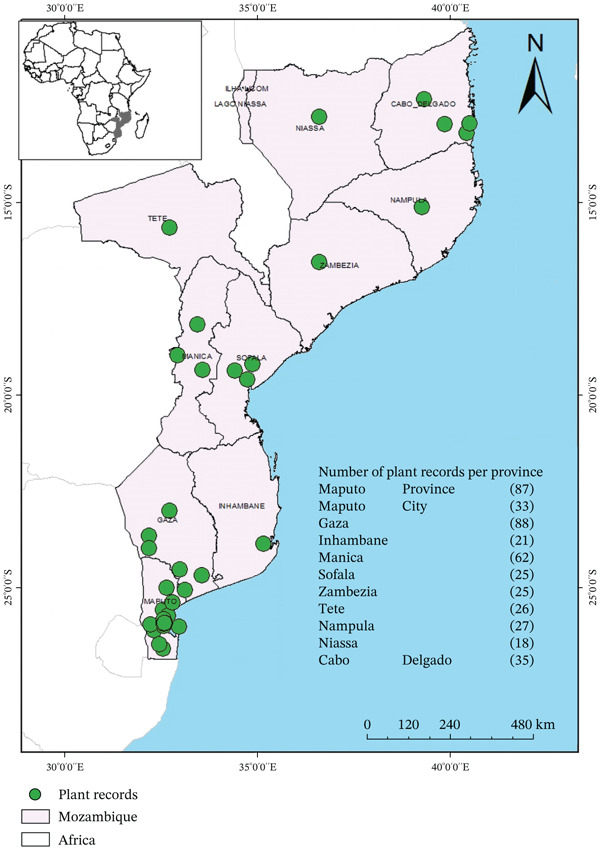
Geographic distribution of records of plants used for the treatment of diarrhea in Mozambique.

As illustrated in Figure 5, records of medicinal plant use for diarrhea treatment are widely distributed across Mozambique, spanning from Maputo to provinces in the southern, central and northern regions of the country. The number of citation records varied across the 12 provinces, with the highest concentrations reported in Gaza (88 records) and Maputo Province (87 records). In contrast, Niassa Province had the fewest records, with 18 citations.

Of the total of 153 plants for which origin data were available, the majority (86.3%, *n* = 132) were native to Mozambique, highlighting the importance of indigenous flora in traditional medicine. Introduced species accounted for 13.7% (*n* = 21). One species, *Aloe marlothii*, cited in multiple studies [35, 40, 42–44], is considered near endemic to Mozambique. In addition, varieties of three species (*Acridocarpus natalitius* var. *linearifolius*, *Dichrostachys cinerea* var. *pubescens* and *Ozoroa obovata* var. *elliptica*) are also classified as near endemic.

Regarding conservation status, IUCN data were available for 106 species. Among these, 94.3% (*n* = 100) were classified as least concern, whereas three species were listed as data deficient. Three species (*Ansellia africana* [65], *Combretum goetzei* [66] and *Warburgia salutaris* [67]) were classified as vulnerable, indicating an elevated risk of extinction in the wild.

## 4. Discussion

This systematic review documents 174 plant species used for the treatment of diarrhea in Mozambique, reflecting the breadth and depth of traditional medical knowledge across the country. This body of knowledge illustrates the long‐standing reliance of local communities on plant‐based remedies for managing gastrointestinal disorders and underscores the relevance of traditional medicine within the national healthcare landscape.

The diversity of sources included in this review highlights the multiple pathways through which ethnobotanical knowledge is recorded, from scientific articles, theses, books, and reports. Despite the extensive documentation compiled, the number of plants identified represents only a fraction of Mozambique′s botanical diversity, which comprises more than 7,000 vascular plant taxa [22].

Temporal trends in publication indicate a decline in documentation efforts after 2010, highlighting a need to reinvigorate ethnobotanical research in Mozambique. In addition, a marked geographic imbalance was observed, with a higher concentration of records from southern provinces, particularly Gaza and Maputo Province. This pattern likely reflects the geographic distribution of research institutions rather than actual differences in traditional medicine use. Expanding research efforts in central and northern Mozambique is essential to ensure a more representative understanding of plant‐based therapeutic practices nationwide.

Roots were the most frequently used plant parts for diarrhea treatment, consistent with previous ethnobotanical studies in Mozambique [28, 34, 35, 38]. Although roots are often valued for their perceived potency, their preferential use raises important sustainability concerns. Root harvesting can impair plant regeneration and threaten population viability, particularly for species already under ecological pressure. Promoting the use of alternative plant parts, such as leaves or bark when pharmacologically appropriate, alongside sustainable harvesting practices, is therefore critical to balancing therapeutic use with conservation.

Preparation methods varied widely, with decoction being the most reported method. This likely reflects its effectiveness in extracting water‐soluble bioactive compounds and facilitating direct gastrointestinal action following oral administration. However, some less common practices, including prechewing remedies for children, herbal enemas and the application of smoke to the anus may pose safety risks due to the potential for infection, irritation, or other adverse reactions.

Most of the documented species were native to Mozambique, emphasizing the reliance on indigenous flora for healthcare. The presence of near‐endemic and vulnerable species highlights the urgency of integrating conservation strategies into traditional medicine practices. Protecting habitats, regulating harvesting, and engaging communities in conservation efforts are essential to ensuring the long‐term availability of these medicinal resources.

The frequent citation of *T. sericea* in traditional medicine for the treatment of diarrhea is consistent with findings from a literature review conducted in South Africa [68], reinforcing its relevance across southern Africa. Beyond its use for diarrhea, ethnobotanical studies in Mozambique report *T. sericea* as a multipurpose medicinal plant employed in the management helminthiases, hemorrhoids, menstrual disorders, female infertility, diabetes, wound healing, stomach pain, and malaria [28, 34, 35, 64].

The antidiarrheal potential of *T. sericea* has been associated with its rich phenolic composition. In particular, compounds such as anolignan B and quercetin‐3‐(2″ galloylrhamnoside) have demonstrated strong antimicrobial activity against *E. coli*, with minimum inhibitory concentrations (MICs) of 0.031 mg/mL and 0.38 mg/mL, respectively [69, 70]. In addition, organic extracts obtained from *T. sericea* showed marked antimicrobial activity against *E. coli* and *Shigella flexneri*, with MIC values of 0.015 mg/mL and 0.04 mg/mL, respectively [69, 71]. These findings provide pharmacological support for its traditional use and highlight the species as a promising source of bioactive compounds for the development of novel antidiarrheal and antimicrobial agents.

*Annona senegalensis*, the second most frequently cited species in this review, is widely recognized for its medicinal importance across Africa. Its use has been reported not only in Mozambique but also in several other African countries [72–80]. In Mozambican traditional medicine, the leaves, bark, and roots of *A. senegalensis* are used to treat a range of conditions, particularly gastrointestinal disorders, respiratory ailments, and sexually transmitted infections [28, 34, 35].

The antidiarrheal effect of *A. senegalensis* has been supported by experimental evidence. In an *in vivo* study, aqueous extracts of the bark and roots reduced the frequency of defecation, inhibited intestinal transit, and decreased intestinal fluid accumulation in rats with castor oil‐induced diarrhea [81]. These results provide a biological basis for its traditional use and suggest that further investigation into its mechanisms of action is warranted. Additional pharmacological studies, including toxicity assessments and clinical trials, would be essential to support the development of evidence‐based therapeutic applications derived from *A. senegalensis* for the management of diarrhea and related gastrointestinal disorders.

## 5. Conclusion

This systematic review provides a comprehensive overview of medicinal plants used to treat diarrhea in Mozambique, revealing a rich diversity of species, preparation methods and traditional practices. Frequently cited plants such as *Terminalia sericea*, *Annona senegalensis*, *Vernonia colorata*, *Ozoroa obovata* and *Sclerocarya birrea* play a central role in traditional diarrhea management and represent promising candidates for further investigation.

The study emphasizes the importance of ethnobotanical knowledge as a resource for identifying bioactive compounds with potential antidiarrheal properties. However, advancing this field requires systematic pharmacological validation, toxicological assessment, and, ultimately, clinical evaluation to ensure safety and efficacy.

Strengthening research infrastructure in Mozambique, including laboratory capacity, training, and funding, is essential to support rigorous investigation of traditional medicinal plants. In parallel, fostering interdisciplinary collaboration among researchers, healthcare professionals, traditional healers, policymakers, and communities will facilitate knowledge exchange and support the integration of validated traditional medicine into healthcare systems.

By combining ethnobotanical knowledge with scientific investigation, this integrated approach offers a pathway toward developing effective, culturally appropriate, and sustainable interventions for diarrhea. Such efforts have the potential not only to improve public health outcomes in Mozambique but also to contribute to global strategies addressing diarrheal disease and antimicrobial resistance.

## Funding

No funding was received for this manuscript.

## Conflicts of Interest

The authors declare no conflicts of interest.

## Supporting Information

Additional supporting information can be found online in the Supporting Information section.

## Supporting information


**Supporting Information 1** PRISMA 2020 Checklist.

**Supporting Information 2** Search strategy.

**Supporting Information 3** List of plants used for diarrhea treatment: examining plant names, botanical families, parts, preparation methods, IUCN status, origin, endemism, citation percentage, and occurrence details.

## Data Availability

All data generated or analyzed during this study are included in this published article and its supporting information files.
