# Supplementary material for: A Systematic Review of Plants Used for the Treatment of Diarrhea in Mozambique
Source: Biomed Res Int. 2026 Mar 8;2026:4132094. doi: 10.1155/bmri/4132094 (PMC12968327; doi:10.1155/bmri/4132094)
Supplement: Supplementary file 2 — Supporting Information 2 Search strategy. [file BMRI-2026-4132094-s001.docx]

**A systematic review of plants used for the treatment of diarrhea in Mozambique**

**Search strategy**

| **April 10, 2024** |
| --- |

Table 1. Search strategy in databases of medicinal plants used for treatment of diarrhea in Mozambique.

| Database | Search terms | Records found |
| --- | --- | --- |
| PubMed | ((Ethnobotany OR Ethnobotanical OR Ethnomedicinal OR Plants) AND (Diarrhea OR diarrhoea)) AND (Mozambique) | 8 |
| ScienceDirect | Diarrhoea OR Diarrhea AND Plants OR "Medicinal plants" OR Ethnobotany AND Mozambique  Title, abstract, keywords: Mozambique AND plants | 25 |
|  | Total | 33 |
