# Supplementary material for: A Systematic Review of Plants Used for the Treatment of Diarrhea in Mozambique
Source: Biomed Res Int. 2026 Mar 8;2026:4132094. doi: 10.1155/bmri/4132094 (PMC12968327; doi:10.1155/bmri/4132094)
Supplement: Supplementary file 3 — Supporting Information 3 List of plants used for diarrhea treatment: examining plant names, botanical families, parts, preparation methods, IUCN status, origin, endemism, citation percentage, and occurrence details. [file BMRI-2026-4132094-s003.docx]

**A SYSTEMATIC REVIEW OF PLANTS USED FOR THE TREATMENT OF DIARRHEA IN MOZAMBIQUE**

Adilência Mataveia^1^, Filomena Barbosa^1^, Sílvia Langa^1^, Custódio Bila^2^, Valeriano Chichava^1^, Natália Ngome^1^, Mércia Inroga^3^, Helena Correia^4^, Delfina Hlashwayo^1^*

^1^ Departamento de Ciências Biológicas, Faculdade de Ciências, Universidade Eduardo Mondlane, Maputo, Mozambique

^2^ Faculdade de Veterinária, Universidade Eduardo Mondlane, Maputo, Mozambique

^3^ Direcção de Pesquisa em Saúde e Bem-Estar, Instituto Nacional de Saúde, Maputo, MISAU, Maputo, Mozambique

^4^ Centro de Investigação e Desenvolvimento em Etnobotânica, Namaacha, Mozambique

[delfinahlashwayo@gmail.com](mailto:delfinahlashwayo@gmail.com) * corresponding author

| List of plants used for diarrhea treatment: examining plant names, botanical families, parts, preparation methods, IUCN status, origin, endemism, citation percentage and occurrence details |
| --- |

| **Scientific Name** | **Vernacular Name(s) in alphabetical order** | **Botanical family** | **Part used** | **Methods of preparation** | **References** | **IUCN status** | **Native or Introduced** | **Endemic status** | **No. of reports citing (%)** | **Location where plants were obtained** |
| --- | --- | --- | --- | --- | --- | --- | --- | --- | --- | --- |
| *Abrus precatorius* L. | Mini-mini, mutitivaroi, sissana | Fabaceae | Roots | Maceration; Make mush | [1, 2] | NR | Native | NR | 2 (5.3%) | Katembe (MC) [1], Muda (MA) [2] |
| *Acanthospermum glabratum* (DC.) Wild | NR | Asteraceae | Whole plant | Decoction | [3] | NR | Introduced | - | 1 (2.6%) | MP and IB [3] |
| *Acridocarpus natalitius* A.Juss. | Mabope, mabhope, mabhophe | Malpighiaceae | Roots | Decoction | [4-7] | Least concern↓ | Native | Near endemic† | 4 (10.5%) | Medicinal plant markets (MC) [5], MP and GZ [7], Chokwé and Bilene (GZ) [6] |
| *Adansonia digitata* L. | Baobab, nonge, ximuhu, ximuvo, ximuwa | Malvaceae | Roots | Maceration | [4, 8, 9] | NR | Native | NR | 3 (7.9%) | Massingir district (GZ) [8], Mecufi (CD) [9] |
| *Adenium multiflorum* Klotzsch | NR | Apocynaceae | NR | NR | [10] | Least concern↓ | Native | NR | 1 (2.6%) | Limpopo National Park (GZ) [10] |
| *Albizia adianthifolia* (Schumach.) W.Wight | Gowana | Fabaceae | Bark | NR | [11] | Least concern | Native | NR | 1 (2.6%) | Matutuíne (MP) [11] |
| *Albizia versicolor* Welw. ex Oliv. | Mugomati, Mutundurulo | Fabaceae | Roots | Maceration | [2] | Least concern | Native | NR | 1 (2.6%) | Muda (MA) [2] |
| *Aloe chabaudii* Schönland | Mangane ya yi tsngo | Asphodelaceae | NR | NR | [12] | NR | Native | NR | 1 (2.6%) | MP and GZ [12] |
| *Aloe marlothii* A.Berger | Aloe vera, Mangana le y kulo, Mangane, Mangane ya yi kulo, Mhanga | Asphodelaceae | Leaves | Decoction | [5, 10, 12-14] | Least concern | Native | Near endemic | 5 (13.2%) | Medicinal plant markets (MC) [5], Magude district (MP) [14], MP and GZ [12], Limpopo National Park (GZ) [10, 13] |
| *Anacardium occidentale* L. | N’khanju, Nkhanju | Anacardiaceae | Roots, stem, leaves | Decoction | [12, 15, 16] | Least concern | Introduced | - | 3 (7.9%) | Inhaca Island (MP) [16], MP [12], Ancuabe district, CD [15] |
| *Ananas comosus* (L.) Merr. | NR | Bromeliaceae | Leaves | Decoction | [17] | NR | Introduced | - | 1 (2.6%) | Boane district (MP) [17] |
| *Ancylobothrys petersiana* Pierre | Didocomela, docomela, documela, gulunje, idocomela, indocomela, inrava, itamuela, mabunze, macara, macava, matatu-bonsu, mautiele, mino, mpire, mtolia, mudocomene, mutiele, mututobunze, muzamera, n’tamungu, rapalala, rava, tamuela, tamunga, tenguela, tengwela, tuela, utewela | Apocynaceae | Roots | Decoction | [18] | NR | Native | NR | 1 (2.6%) | MP, GZ, IB, SO, ZB, T, NA [18] |
| *Annona muricata* L. | NR | Annonaceae | Leaves | Decoction | [17] | Least concern | Introduced | - | 1 (2.6%) | Boane district (MP) [17] |
| *Annona senegalensis* Pers. (Synonym reported: *Annona senegalensis* subsp. *senegalensis*) | Anoneira cafreal, ata do mato, ata silvestre, budzo, chipamsuro, chofa, dirova, dirova, iembe, iépe, iépe, ingopfa, intope, jenge, jenge, lembe, lembe, lembe, libudzo, litope, llolo, m’lembe, m’posa, m’poza, m’rrova, maièpe, marofa, maronfa, marôpa, maropfa, mazova, melembe, merronfa, metópè, metopetove, mezof, mroro, muanaquire, muculati, muiebe, muiebê, muiembe, muiepe, muiepè, muiépe, muièpe, mujebe, mulemba, mulembe, mulolo, mulukama, muniznge, munroro, mupuza, muroro, murôro, murrepe, murronfa, murrôro, mutsomboro, n’djofa, ncalati, ndzopfa, nrepe, nrepe, nsasa, nthyonpha, ntopo, répè, rhompha, rhompifa, rofa, rôfa, rompfa, rompha, rompifa, ronfa, ropfa, rova, thopfa | Annonaceae | Roots (including root bark), stem, leaves | Decoction; Maceration; Make porridge with maceration; Char and add to food | [4, 7, 10, 15, 17, 19-25] | Least concern | Native | NR | 12 (31.6%) | MC [24], Boane district (MP) [17], Matutuíne district (MP) [19, 21], MP [23], Manica district (MA) [22], MP and GZ [7, 20], Limpopo National Park (GZ) [10], MP, GZ, IB, SO, MA, ZB, T, NA, CD, NI [25], Ancuabe district (CD) [15] |
| *Ansellia africana* Lindl. | NR | Orchidaceae | NR | NR | [10] | Vulnerable↓ | Native | NR | 1 (2.6%) | Limpopo National Park (GZ) [10] |
| *Antidesma venosum* E.Mey. ex Tul. | Txungi, utsungui | Phyllanthaceae | Leaves, roots | Decoction (can include mixtures with other plants [16]) | [16, 24, 26] | Least concern | Native | NR | 3 (7.9%) | Marracuene district (MP) [26], Inhaca Island (MP) [16], MC [24] |
| *Artabotrys brachypetalus* Benth. | Tita | Annonaceae | Roots, bark | Maceration | [26] | Least concern | Native | NR | 1 (2.6%) | Marracuene district (MP) [26] |
| *Boscia albitrunca* (Burch.) Gilg & Benedict | Nxunkutso, xikutse, xikutsi, xikutso, xikutsu | Capparaceae | Leaves | Infusion after crushing | [8, 13] | Least concern | Native | NR | 2 (5.3%) | Limpopo National Park (GZ) [13], Massingir district (GZ) [8] |
| *Boscia salicifolia* Oliv. | Calajuju, canungonungo, inhege, inhenge, inhézi, khala-tchulu, mapasserua, melherere, metosi, monapo, munhanja, munhensa, munhenza, murijamezu, muruza, mutuconuco, mutugungu, namecapuane, nhendze, nhenza, nyendzi, tombogaga | Capparaceae | Roots | Decoction (with roots of *Thilachium africanum* Lour.) | [27] | Least concern | Native | NR | 1 (2.6%) | SO, MA, ZB, T, NA, CD, NI [27] |
| *Brachylaena discolor* DC. (Synonym reported: *Brachylaena discolor* subsp. *discolor*) | Mpalha, palhakufa | Asteraceae | Roots, leaves | Decoction; Infusion; Maceration | [16, 28] | Least concern | Native | NR | 2 (5.3%) | Inhaca Island (MP) [16], Marracuene and Boane (MP) [28] |
| *Brackenridgea zanguebarica* Oliv. | Mumino | Ochnaceae | Roots, bark | Maceration | [2] | NR | Native | NR | 1 (2.6%) | Muda (MA) [2] |
| *Brexia madagascariensis* (Lam.) Thouars ex Ker Gawl. | Ungamunga | Celastraceae | Bark | Decoction | [16] | Least concern↓ | Native | NR | 1 (2.6%) | Inhaca Island (MP) [16] |
| *Bridelia cathartica* Bertol. | Balatangati, munwangate, munwangati, thlanthlangati, thlathlangate | Phyllanthaceae | Leaves, roots | Decoction | [7, 12, 19] | Least concern | Native | NR | 3 (7.9%) | Matutuíne district (MP) [19], Boane district (MP) [14], MP [12], MP and GZ [7] |
| *Burkea africana* Hook. | NR | Fabaceae | Roots, stem, leaves | NR | [15] | Least concern↓ | Native | NR | 1 (2.6%) | Ancuabe district (CD) [15] |
| *Calotropis gigantea* (L.) Dryand. | Nseda, séta | Apocynaceae | Root bark | Powder; Tincture | [18] | NR | Introduced | - | 1 (2.6%) | MP, CD [18] |
| *Carica papaya* L. | Mupapaya | Caricaceae | Leaves, stem, roots | Maceration | [2, 15] | Data deficient↓ | Introduced | - | 2 (5.3%) | Muda (MA) [2], Ancuabe district (CD) [15] |
| *Carissa spinarum* L. | NR | Apocynaceae | NR | NR | [10] | Least concern | Native | NR | 1 (2.6%) | Limpopo National Park (GZ) [10] |
| *Cassia abbreviata* Oliv. | Lumanyama, murumanhama, numanhama | Fabaceae | Roots (including root bark), stem, leaves, bark | Decoction; Maceration | [2, 6-8, 15] | Least concern | Native | NR | 5 (13.2%) | MP and GZ [7], Chokwé and Bilene (GZ) [6], Massingir district (GZ) [8], Muda (MA) [2], Ancuabe district (CD) [15] |
| *Catharanthus roseus* (L.) G.Don | Beijo-de-mulata, chicaudha, felor, flor, guiana, inchamba, mafilore akubassa, maflore akubassa, mudaca nhoca, nyoka, xifilorana, xifilorane, xiflorana, xiflorane | Apocynaceae | Whole plant, roots | Decoction | [1, 9, 14, 18, 19, 29] | NR | Introduced | - | 6 (15.8%) | Katembe (MC) [1], Matutuíne district (MP) [19], Magude district (MP) [14], Homoine district, IB [29], MP, GZ, MA, NI [18], Nanchukuro, CD [9] |
| *Choritaenia capensis* Benth. (Synonym reported: *Pappea capensis* Sond. & Harv*.*) | NR | Apiaceae | NR | NR | [10] | Least concern | Native | NR | 1 (2.6%) | Limpopo National Park (GZ) [10] |
| *Cissampelos mucronata* A.Rich. | NR | Menispermeaceae | NR | NR | [4] | NR | Native | NR | 1 (2.6%) | NR [4] |
| *Cissus cornifolia* Planch. | NR | Vitaceae | NR | NR | [10] | NR | Native | NR | 1 (2.6%) | Limpopo National Park (GZ) [10] |
| *Cissus rotundifolia* Vahl | Pangalatani | Vitaceae | Roots | NR | [13] | NR | Native | NR | 1 (2.6%) | Limpopo National Park (GZ) [13] |
| *Citrus aurantiifolia* (Christm.) Swingle | Limão | Rutaceae | NR | NR | [9] | NR | NR | NR | 1 (2.6%) | Zimpeto (MC) [9] |
| *Citrus limon* (L.) Osbeck | Mchugia, mdimo, titela | Rutaceae | Roots | NR | [22] | NR | NR | NR | 1 (2.6%) | Manica district (MA) [22] |
| *Cladostemon kirkii* (Oliv.) pax e Gilg | Mahukwe, miashicombo, munhabsi-combo, nhamurekwane, sizumbu | Capparaceae | Roots | Decoction; Infusion; Maceration | [2, 6, 10, 16, 19] | Least concern | Native | NR | 5 (13.2%) | Matutuíne district (MP) [19], Inhaca Island (MP) [16], Chokwé and Bilene (GZ) [6], Limpopo National Park (GZ) [10], Muda (MA) [2], |
| *Clausena* sp. (Name reported *Clausena etiopica –* unchecked) | Muthusi | Rutaceae | NR | NR | [30] | * | * | * | 1 (2.6%) | Licuati (MP) [30] |
| *Cleome angustifolia* Forssk*.* (Synonym reported: *Cleome angustifolia* subsp. *petersiana* (Klotzsch) Kers) | NR | Cleomaceae | Seeds | Infusion | [31] | NR | Native | NR | 1 (2.6%) | MP and GZ [31] |
| *Cocculus hirsutus* (L.) W.Theob. | Mussoropotu | Menispermaceae | Roots, leaves | Maceration | [2] | NR | Native | NR | 1 (2.6%) | Muda (MA) [2] |
| *Cola* sp. | Chitswati, mutiti | Malvaceae | Leaves | NR | [22] | * | * | * | 1 (2.6%) | Manica district (MA) [22] |
| *Colophospermum mopane* (Benth.) Leonard | Gungwa, mesanya, mopane, nxanati, nxanatsi, xanatsi | Fabaceae | Leaves, stem, bark; Mix of stem and leaves | Decoction; Infusion; Maceration (after grinding) | [8, 13] | Least concern↓ | Native | NR | 2 (5.3%) | Limpopo National Park (GZ) [13], Massingir district (GZ) [8] |
| *Combretum adenogonium* Steud. ex A.Rich. (Synonym reported: *Combretum fragrans* F.Hoffm.) | Chinama, ètchinama, èxinama, fiti, ginama, ginama, inama, m’findo, m’pfiti, mugodo, mutucatura, nacuena, nama, nnama, shipoza, tinama, ubo, ubu, upo, xiposa | Combretaceae | Roots | Add to porridge after grinding and adding *Hoslundia opposita* and *Tricalysia jasminiflora* roots | [3] | Least concern | Native | NR | 1 (2.6%) | SO, MA, ZB, T, NA, CB, NI [3] |
| *Combretum apiculatum* Sond. | NR | Combretaceae | NR | NR | [10] | Least concern | Native | NR | 1 (2.6%) | Limpopo National Park (GZ) [10] |
| *Combretum goetzei* Engl. & Diels | Ngochi | Combretaceae | Roots | Make mush | [2] | Vulnerable | Native | NR | 1 (2.6%) | Muda (MA) [2] |
| *Combretum imberbe* Wawra | Mlanane, mocôda, mocossa, mocoza, mondu, mondu-n-hlovu, mondzo, môndzo, monkôsso, monso, monzo, monzou, msimbiti, mucádi, mucado, mucando, mulangani, munangar, munangari, nacuada, nacucuni, nangale, nangali, nhangare, t’simbiti, xsimbite | Combretaceae | Bark | Add to corn porridge after grinding | [3, 10] | Least concern↓ | Native | NR | 2 (5.3%) | Limpopo National Park (GZ) [10], MP, GZ, IB, SO, MA, ZB, T, NA [3] |
| *Combretum microphyllum* Klotzsch | Babangoena, bambangwena, baubaubauguena, cotamo, duva, ecotamo, funté, hlaba-konkonya, m’kotamu, mhlalavan, mirrame, mucutemo, muílatubili, muirrama, mumbambanguene, mumbambanguene pfunte, muriacololo, nhacuruerue, nkotamo, nkotamu, pfunte, pfunté, pfute, ripari, seluba, tuetue, twetwe | Combretaceae | Leaves, roots | Maceration | [3, 8, 10] | NR | Native | NR | 3 (7.9%) | MP, GZ, IB, SO, MA, ZB, T, NA, CB, NI [3], Massingir district (GZ) [8], Limpopo National Park (GZ) [10] |
| *Combretum molle* R.Br. ex G.Don | Cadâli, cagunguni, chicucudze, chicuiche, chiquche, chiquiche, chisunliti, civondzuana, ehubo, eumbo, fitidonga, fitigonco, ginama, giniama, hupo, inama, kankunguni wa m’pongo, katumba, m’cumbunhi, mbondomnyama, mifiti, miquitchi, mpugunha, mugoge, mulala, mulama, mutsinguidzi, pupunha, pupunha, secôônhôto, shikukutse, siquiche, tsinama, xicucutce, xicucutse, xicucutsi, xikukutse, xikukutso, xikwiri, xiwondzwana, | Combretaceae | Roots | Decoction; Make corn porridge with decoction | [3-7, 10, 12] | Least concern | Native | NR | 7 (18.4%) | Medicinal plant markets (MC) [5], MP and GZ [12] [7], Chokwé and Bilene (GZ) [6], Limpopo National Park (GZ) [10], MP, GZ, IB, SO, MA, ZB, T, NA, CD, NI [3] |
| *Combretum mossambicense* Engl. | Bambamgwene, bombanguena, chicula’ussico, ecotamo, fiti, funté, fute, futé, kotamo, mcotano, mwatama, tuétué | Combretaceae | Roots, leaves | Maceration (*Albizia harveyi* can be added when roots are utilized, or not in case of leaves) | [3, 8, 10] | Least concern | Native | NR | 3 (7.9%) | MP, GZ, IB, SO, MA, ZB, T, NA [3], Massingir district (GZ) [8], Limpopo National Park (GZ) [10] |
| *Combretum padoides* Engl. & Diels | Chikwizi, xikwitsi, | Combretaceae | Roots | Decoction | [30] | Least concern | Native | NR | 1 (2.6%) | Licuati (MP) [30] |
| *Combretum* sp. | Fufu, nhathelo, xiwonzuane | Combretaceae | NR | NR | [12] | * | * | * | 1 (2.6%) | MP [12] |
| *Combretum zeyheri* Sond. | N'fufu | Combretaceae | NR | NR | [10, 12] | Least concern | Native | NR | 2 (5.3%) | MP [12], Limpopo National Park (GZ) [10] |
| *Commiphora africana* (Synonym reported: *Commelina benghalensis* Forssk.) | Khonfanhe | Commelinaceae | NR | NR | [10, 32] | Least concern | Native | NR | 2 (5.3%) | Magude district (MP) [32], Limpopo National Park (GZ) [10] |
| *Cordia monoica* Roxb. | NR | Boraginaceae | NR | NR | [10] | Least concern | Native | NR | 1 (2.6%) | Limpopo National Park (GZ) [10] |
| *Crossopteryx febrifuga* Benth. | Chicobengua, mucobengua | Rubiaceae | Roots, leaves, bark | Maceration | [2] | Least concern | Native | NR | 1 (2.6%) | Muda (MA) [2] |
| *Cucumis zeyheri* Sond. | Chihacaiane, xihakahani, xiyakayani | Curcubitaceae | Leaves | Decoction; Maceration | [8, 10] | NR | Native | NR | 2 (5.3%) | Massingir district (GZ) [8], Limpopo National Park (GZ) [10] |
| *Cyperus articulatus* L. | Dzudzulutana, tindzau, | Cyperaceae | Roots | The mother chews and feeds to the child, while applying a small amount to the child's abdomen | [33] | Least concern | Native | NR | 1 (2.6%) | MP, GZ, T, CD [33] |
| *Dichrostachys cinerea* (L.) Wight & Arn. | Chinjonjonjo, chinteni | Fabaceae | Roots | Maceration | [2] | Least concern | Native | Near Endemic† | 1 (2.6%) | Muda (MA) [2] |
| *Diospyros galpinii* (Hiern) De Winter | Chiconboti | Ebenaceae | Roots | Maceration | [2] | Least concern | NR | NR | 1 (2.6%) | Muda (MA) [2] |
| *Diospyros mespiliformis* Hochst. ex A.DC. | NR | Ebenaceae | NR | NR | [10] | Least concern | Native | NR | 1 (2.6%) | Limpopo National Park (GZ) [10] |
| *Diplorhynchus condylocarpon* (Müll.Arg.) Pichon | M’toa | Apocynaceae | Sap | Ingestion | [2] | Least concern | Native | NR | 1 (2.6%) | Muda (MA) [2] |
| *Dodonaea viscosa* Jacq. | Tchotila | Sapindaceae | NR | NR | [12] | Least concern | Native | NR | 1 (2.6%) | Limpopo National Park (GZ) [12] |
| *Dombeya rotundifolia* (Hochst.) Planch. | NR | Malvaceae | NR | NR | [10] | Least concern | Native | NR | 1 (2.6%) | Limpopo National Park (GZ) [10] |
| *Drimia altissima* (L.f.) Ker Gawl. (Synonym reported: *Urginea altissima* (L.f.) Baker) | Munhaca, xicatsana | Asparagaceae | Bulb (referred to as Tuber) | NR | [14, 32] | NR | Native | NR | 2 (5.3%) | Magude district (MP) [32], Boane and Magude districts (MP) [14] |
| *Dysphania ambrosioides* (L.) Mosyakin & Clemants (Synonym reported *Chenopodium ambrosioides* L.) | Kanuka uncono | Amaranthaceae | Leaves | Infusion | [3] | NR | Introduced | - | 1 (2.6%) | MP, GZ, SO, T, NA, NI [3] |
| *Ehretia amoena* Klotzsch (Synonym reported: *Ehretia stuhlmanni* Gürke) | Guaracuasho, mulavilavi, namulavilavi | Boraginaceae | Bark, roots | Maceration; Make porridge with corn flour after grinding with *Dialium holtzii* | [2, 12, 27] | Least concern | Native | NR | 3 (7.9%) | MP [12], Muda (MA) [2], ZB, NA, CD [27] |
| *Elachyptera parvifolia* (Oliv.) N.Hallé (  Synonym reported: *Hippocratea parvifolia*  Oliv*.*) | Lurro, murama, sangueleiti | Celastraceae | Roots | Infusion | [3] | NR | Native | NR | 1 (2.6%) | GA, SO, T, NA, CD, NI [3] |
| *Elaeodendron schlechterianum* Loes. | NR | Celastraceae | NR | NR | [10] | Least concern | Native | NR | 1 (2.6%) | Limpopo National Park (GZ) [10] |
| *Elephantorrhiza elephantina* (Burch.) Skeels | Nyassitane, xivurai | Fabaceae | Roots | Decoction; Maceration | [4-7, 10] | NR | Native | NR | 5 (13.2%) | Medicinal plant markets MC [5], Chokwé and Bilene (GZ) [6], MP and GZ [7], Limpopo National Park (GZ) [10] |
| *Erianthemum dregei* Tiegh. | Pakama | Loranthaceae | NR | NR | [13] | NR | Native | NR | 1 (2.6%) | Limpopo National Park (GZ) [13] |
| *Erythroxylum emarginatum* Thonn. | NR | Erythroxylaceae | Roots, stem, leaves | NR | [15] | Least concern | Native | NR | 2 (5.3%) | Ancuabe district (CD) [15] |
| *Euclea divinorum* Hiern | NR | Ebenaceae | NR | NR | [10] | Least concern | Native | NR | 1 (2.6%) | Limpopo National Park (GZ) [10] |
| *Euphorbia hirta* L. | Chinhamucaca de flor | Euphorbiaceae | Roots | Make mush | [2] | NR | Introduced | - | 1 (2.6%) | Muda (MA) [2] |
| *Ficus sur* Forssk. | NR | Moraceae | NR | NR | [10] | Least concern | Native | NR | 1 (2.6%) | Limpopo National Park (GZ) [10] |
| *Flacourtia indica* (Burm.f.) Merr. | Mundiduè | Salicaceae | Leaves | NR | [31] | Least concern | Native | NR | 1 (2.6%) | IB, MA, NA, NI, SO, T, ZB [31] |
| *Flueggea virosa* (Roxb. ex Willd.) Royle | NR | Phyllanthaceae | NR | NR | [10] | Least concern | Native | NR | 1 (2.6%) | Limpopo National Park (GZ) [10] |
| *Garcinia livingstonei* T.Anderson | Bimbe, himbe, mahimbe, mbimbe, phimbi, vimbe | Clusiaceae | Roots, stem, bark | Decoction | [4, 5, 7, 10-12, 14, 26] | Least concern | Native | NR | 8 (21.1%) | Medicinal plant markets (MC) [5], Marracuene district (MP) [26], Magude district (MP) [14], Matutuíne (MP) [11], MP and GZ [7, 12], Limpopo National Park (GZ) [10] |
| *Gardenia cornuta* Hemsl. | NR | Rubiaceae | Roots | NR | [20] | Least concern | Native | NR | 1 (2.6%) | MP and GZ [20] |
| *Gardenia ternifolia* Schumach. & Thonn. | Chintarara | Rubiaceae | Roots, bark | Make mush | [2] | Least concern | Native | NR | 1 (2.6%) | Muda (MA) [2] |
| *Gardenia volkensii* K.Schum. (Synonym reported: *Gardenia volkensii* subsp. *volkensii*) | Chitzalala | Rubiaceae | Fruit | Maceration | [31] | Least concern | Native | NR | 1 (2.6%) | MA [31] |
| *Gladiolus dalenii* Van Geel | Halaingua | Iridaceae | Roots, bulb | Decoction; Infusion | [4, 19] | NR | Native | NR | 2 (5.3%) | Matutuíne district (MP) [19] |
| *Gladiolus* sp. | Halahingwa, mugathandela | Iridaceae | Roots, bulb | Decoction | [5, 12] | * | * | * | 2 (5.3%) | Medicinal plant markets (MC) [5], GZ [12] |
| *Gossypium herbaceum* L. | Thondge ya kwati | Malvaceae | NR | NR | [12] | Data deficient | Introduced | - | 1 (2.6%) | MP [12] |
| *Grewia monticola* Sond. | Nsihana, nsihani, nsiphane | Malvaceae | Roots | Decoction | [8] | Least concern | Native | NR | 1 (2.6%) | Massingir district (GZ) [8] |
| *Gymnosporia buxifolia* (L.) Szyszył. | NR | Celastraceae | NR | NR | [10] | Least concern | Native | NR | 1 (2.6%) | Limpopo National Park (GZ) [10] |
| *Gymnosporia heterophylla* Loes. (Synonym reported: *Maytenus heterophylla* (Eckl. & Zeyh.) N.Robson) | Chichangua, dimbazou, khala-mavu, libatzondze, muiua, mutunga-macheche, n’qokola, xihlangua, xilhangua | Celastraceae | Roots, leaves | Decoction; Infusion; Maceration | [2, 3, 5, 30] | Least concern | Native | NR | 4 (10.5%) | Medicinal plant markets (MC) [5] Licuati, MP [30], MP, GZ, IB, SO, MA, ZB, T, NA, CB [3], Muda (MA) [2] |
| *Gymnosporia senegalensis* Loes. (Synonym reported: *Maytenus senegalensis* (Lam.) Exell) | Bobué, chichanga, chichangua, chilhangua, chilhaungua, chixangua, chixangua, cungamacheze, fogolia, m’tocoma, mitocoma, muia, mutuluca, mutumbotumbo, mutunga-macheche, nacôtocôto, napidji, nhaquitofororo, patchocolo, sucameno, tambanzato, tomatsatu, tombanzato, tombassato, tombatsato, tsucamano, xichângue xilangua, xilhangua, xixangua | Celastraceae | Roots, leaves | Infusion | [3, 4, 8, 14, 26] [10, 31] | Least concern | Native | NR | 7 (18.4%) | Marracuene district (MP) [26], Magude district (MP) [14], Massingir district (GZ) [8], GZ, IB [31], Limpopo National Park (GZ) [10], MP, GZ, IB, SO, MA, ZB, T, NA, CD, NI [3] |
| *Hagenia abyssinica* (Bruce) J.F.Gmel. | NR | Rosaceae | Roots, stem, leaves | NR | [15] | Least concern | NR | NR | 1 (2.6%) | Ancuabe district (CD) [15] |
| *Helichrysum kraussii* Sch.Bip. | Chamba-chássuro, chirimbyati, chiringuati , chisimbati, chizimbzati, shiriungati, sthambu, xirhimbswati | Asteraceae | Roots | Decoction | [6, 33] | NR | Native | NR | 2 (5.3%) | Chokwé and Bilene (GZ) [6], MP, GZ, IB, SO, MA, NA [33] |
| *Hugonia orientalis* Engl. | Congulutamute | Linaceae | Roots | Decoction | [5] | Least concern | Native | NR | 1 (2.6%) | Medicinal plant markets (MC) [5] |
| *Hymenocardia acida* Tul. | Chitongua | Phyllanthaceae | Sap from bark | Ingestion after mixing with water | [34] | Least concern | Native | NR | 1 (2.6%) | Macate district (MA) [34] |
| *Hypoxis hemerocallidea* Fisch., C.A.Mey. & Avé-Lall. | Batata africana, xirangabwana | Hypoxidaceae | Corm | Decoction; Maceration | [5, 7] | NR | Native | NR | 2 (5.3%) | Medicinal plant markets (MC) [5], MP and GZ [7] |
| *Hypoxis* sp, | Chirangaboana | Hypoxidaceae | NR | NR | [12] | * | * | * | 1 (2.6%) | MP [12] |
| *Kedrostis* sp. | Dema, dema-amarelo, dema la kupshuka | Curcubitaceae | Roots | Decoction; Maceration | [5, 12] | * | * | * | 2 (5.3%) | Medicinal plant markets (MC) [5], GZ [12] |
| *Kigelia africana* (Lam.) Benth. | Fongosi, fungosi, fungura, fungurre, litandi, mluku-tungwa, mouk-tuk, mpfungula, mpfungura, murrucurruco, muúmo, muvunguti, mvunguti, ntaandi, nvumbo, pfungura, togo-togo, umtuberubu, vongute, vungute, vunguti, | Bignoniaceae | Bark | Decoction | [4, 11, 26, 27] | Least concern | Native | NR | 4 (10.5%) | Marracuene district (MP) [26], Matutuíne, MP [11], MP, GZ, SO, MA, ZB, T, NA, CD, NI [27] |
| *Landolphia* sp. | Nhawutsulwane | Apocynaceae | NR | NR | [29] | * | * | * | 1 (2.6%) | Homoine district (IB) [29] |
| *Lannea discolor* Engl. | Chumbo, mumbo, xiumbucanhane | Anacardiaceae | Roots | Make mush | [2, 12] | Least concern | Native | NR | 2 (5.3%) | MP [12], Muda (MA) [2] |
| *Lannea edulis* Engl. (Synonym reported: *Lannea edulis* var. *edulis*) | Diacamba, inácua, nˊkumbagumba, tchatambalala | Anacardiaceae | Roots, stem (stalk) | Make mush with a spoon of crushed herb; Make porridge with a one-spoon infusion | [23, 25] | NR | Native | NR | 2 (5.3%) | MP [23], MP, MA, ZB, T, NA [25] |
| *Lannea schweinfurthii* Engl. | Chimungumango, chinungo, chinungumafe, chinungumafi, munganikomo, xihumbunkany, ximbucanyi, xinungu, xivombo nkanyi | Anacardiaceae | Bark | Decoction | [6, 8] | NR | Native† | NR | 2 (5.3%) | Chokwé and Bilene (GZ) [6], Massingir district (GZ) [8] |
| *Lannea schweinfurthii* var. *stuhlmannii* (Engl.) Kokwaro (Synonym reported: *Lannea stuhlmannii* (Engl.) Eyles) | Canhupo, chebombocanho, chikumbunkanyi, chimbocanho, chimbucanhi, chimbukani, chimbukanji, chimucanho, chiumbocanhe, chumbocanho, jambirre branco, m’coco, m’poxa, m’suoto, mfula, mubsototo, mucototo, mugano, munganicomo, nhetchecapa, psulussa, schutoto, shimbucanhe, silumelagolane, simuili, sutoto, tchirussa, xihumbonkanye | Anacardiaceae | Roots, bark | Decoction | [23, 25] | NR | Native | NR | 2 (5.3%) | MP, GZ, IB, SO, MA, ZB, T, NA, CD, NI [25], MP [23] |
| *Lantana camara* L. | Chimunhamunhane, ximunhuamunhuana | Verbenaceae | Roots | Decoction (can be mixed with *Strychnos spinosa* [1]) | [1, 12] | NR | Introduced | - | 2 (5.3%) | Katembe (MC) [1], GZ [12] |
| *Lathyrus oleraceus* Lam. (Synonym reported: *Pisum sativum* L.) | NR | Fabaceae | Roots, stem, leaves | NR | [15] | NR | Introduced | - | 1 (2.6%) | Ancuabe district (CD) [15] |
| *Lippia javanica* (Burm.f.) Spreng. | Mussani | Verbenaceae | Root | Decoction | [2] | NR | Native | NR | 1 (2.6%) | Muda (MA) [2] |
| *Maclura africana* (Bureau) Corner (Synonym reported: *Cardiogyne africana*  Bureau) | Mpumbulu, pumbulo, pumbulu | Moraceae | Roots, leaves | Prepare a decoction for oral consumption and for use as an enema (mixtures may be added [1]) | [1, 29] [24] | Least concern | Native | NR | 3 (7.9%) | Katembe (MC) [1], MC [24], Homoine district, IB [29] |
| *Maerua angolensis* DC. | Xinhimanamuri | Capparaceae | Roots, leaves | NR | [13] | Least concern | Native | NR | 1 (2.6%) | Limpopo National Park (GZ) [13] |
| *Maerua parvifolia* Pax | Nongonoko | Capparaceae | Roots | Decoction | [8] | Least concern | Native | NR | 1 (2.6%) | Massingir district (GZ) [8] |
| *Mangifera indica* L. | Manga, mangueira | Anacardiaceae | Seed kernel, Bark | Decoction; Maceration (after grinding) | [9, 17, 26] | Data deficient | Introduced | - | 3 (7.9%) | Marracuene district (MP) [26], Matutuíne district (MP) [9], Boane district (MP) [17] |
| *Margaretta rosea* Oliv. | Chinhamucaca | Apocynaceae | Roots | Maceration | [34] | NR | Native | NR | 1 (2.6%) | Macate district, MA [34] |
| *Maytenus* sp. | Waguvaviro, xixangua | Celastraceae | Stem bark | Decoction | [30, 32] | * | * | * | 2 (5.3%) | Magude district (MP) [32], Licuati (MP) [30] |
| *Melia azedarach* L. | Seringa | Meliaceae | NR | NR | [4, 14] | Least concern | Introduced | - | 2 (5.3%) | Boane and Magude districts (MP) [14] |
| *Moringa oleifera* Lam. | Moringa | Moringaceae | Roots | Decoction | [17] | Least concern | Introduced | - | 1 (2.6%) | Boane district (MP) [17] |
| *Morus alba* L. | Amore, mushongo | Moraceae | Roots | Maceration | [2] | NR | Introduced | - | 1 (2.6%) | Muda (MA) [2] |
| *Nicotiana tabacum* L. | NR | Solanaceae | NR | NR | [14] | NR | Introduced | - | 1 (2.6%) | Boane district (MP) [14] |
| *Ochna natalitia* Walp. | Machanganisso | Ochnaceae | Roots | Decoction | [24] | Least concern | Native | NR | 1 (2.6%) | MC [24] |
| *Ocimum gratissimum L.* (Synonym reported: *Ocimum gratissimum* var. *gratissimum*) | Chinuanúa | Lamiaceae | NR | Infusion | [31] | NR | Native | NR | 1 (2.6%) | MA [31] |
| *Oldenlandia* sp. | Mudahumo | Rubiaceae | Whole plant | Decoction | [19] | * | * | * | 1 (2.6%) | Matutuíne district (MP) [19] |
| *Ozoroa insignis* subsp. *reticulata* (Baker f.) J.B.Gillett (Synonym reported: *Ozoroa reticulata* (Baker f.) R.Fern. & A.Fern.) | Cataossaro, chirenje, mudabikeni, mundungu | Anacardiaceae | Roots, leaves | Maceration | [2] | NR ‡ | Native | NR | 1 (2.6%) | Muda (MA) [2] |
| *Ozoroa obovata* (Oliv.) R.Fern. & A.Fern. | Catançaro, cataussarro, chifissa, chifissa, chifuka, chimafane, chimungumango, chincandue, chinogamafe, chinungo, chinungumafe, chinungumafi, chinungumafo, chiteta, inhangambe, intsassa, manicabi, mecalape, mucacambi, mudjile, musasato, nachicuanga , nacurrapiu, namapuite, namicuri, namucacabi, nicarraga, niharapua, niharrapua, ntoto, xifitho, xifucamafi, xifuga, ximafamafane, ximafana, ximungomafu, xinungu, xinungumafi | Anacardiaceae | Roots, leaves, stem bark | Decoction; Maceration (can be mixed with other plants [1, 16]) | [1, 4, 6, 9, 10, 12, 23, 25, 34] | Least concern | Native | Near endemic † | 9 (23.7%) | Katembe (MC) [1], MP [23], Matutuíne district (MP) [9], MP [12], Inhaca Island (MP) [16], Chokwé and Bilene (GZ) [6], Limpopo National Park (GZ) [10], MP, GZ, IB, SO, MA, ZB, T, NA, CD [25], Macate district, MA [34] |
| *Parthenium* sp. | NR | Asteraceae | NR | NR | [14] | * | * | * | 1 (2.6%) | Boane district (MP) [14] |
| *Peltophorum africanum* Sond. | Goana dzanhaca, maxuvana, mphungancomo | Fabaceae | Roots, bark | Decoction (can be mixed with *Terminalia* [1]) | [1, 10, 12] | Least concern | Native | NR | 3 (7.9%) | Katembe (MC) [1], MP [12], Limpopo National Park (GZ) [10] |
| *Phyllanthus reticulatus* Poir. | Tetenha | Phyllanthaceae | Roots | Decoction | [5] | Least concern | Native | NR | 1 (2.6%) | Medicinal plant markets (MC) [5] |
| *Pluchea dioscoridis* (L.) DC. | Fitifiti, m’bvumbvu, m’fuia, mbvumbvu, munvunvu, navatha, vimba | Asteraceae | Roots, leaves | Maceration (additional details: Cut roots together with *Paederia foetida* root, grind and macerate together with 2 small crabs inside the shell of a big snail; Grind the leaves together with *Indigofera hirsuta* leaves and macerate) | [33] | NR | Native | NR | 1 (2.6%) | MP, GZ, SO, ZB, T, NA, CD [33] |
| *Pseudolachnostylis maprouneifolia* Pax | NR | Phyllanthaceae | NR | NR | [10] | Least concern | Native† | NR | 1 (2.6%) | Limpopo National Park (GZ) [10] |
| *Psidium guajava* L. | Goiaba, guajava, mdau, mguava, mpherhwa, nhacazizi, pejua, pherwa | Myrtaceae | Roots, fruit (referred to as bulb), stem, leaves | Decoction | [6, 9, 12, 16, 22] [15] | Least concern | Introduced | - | 6 (15.8%) | Inhaca Island (MP) [16], Chokwé and Bilene (GZ) [6], GZ [12], Manica district (MA) [22], Dondo (SO) [9], Ancuabe district (CD) [15] |
| *Pterocarpus angolensis* DC. | NR | Fabaceae | NR | NR | [10] | Least concern↓ | Native | NR | 1 (2.6%) | Limpopo National Park (GZ) [10] |
| *Rhoicissus revoilii* Planch. | Dambacerera | Vitaceae | Roots | Maceration | [2] | Least concern | Native | NR | 1 (2.6%) | Muda (MA) [2] |
| *Rhus* sp. | NR | Anacardiaceae | Roots | Decoction (add *Psidium guajava* and *Terminalia sericea*) | [16] | * | * | * | 1 (2.6%) | Inhaca Island (MP) [16] |
| *Rhynchosia sublobata* (Schumach.) Meikle | Munhachiropa, mupeta, nhaxiropa | Fabaceae | Roots | Make mush | [2] | NR | Native | NR | 1 (2.6%) | Muda (MA) [2] |
| *Rourea coccinea* subsp. *boiviniana* (Baill.) Jongkind (Synonym reported: *Byrsocarpus boivinianus* (Baill.) Baill.) | Churo-churo, namonamo, namunamu, napo | Connaraceae | Roots | Decoction | [33] | NR ‡ | Native | NR | 1 (2.6%) | ZB, NA, CD [33] |
| *Rourea orientalis* Baill. (Synonym reported: *Byrsocarpus orientalis* Baill*.*) | Chipangara, chissamba-vacalanga, imiomio, Inhangolo, m’purrunha, m’purunho, mediburana, meprunha, muitho, Munhadoz-warozwa, muprunha, muprunho, muziriri, n’morumoro, namonamo, namo-namo, namu-namu, nnamunamu, P. de macaco, punho muancani, punho mulupati, purunha, sam-baucaranga, samange | Connaraceae | Bark, leaves, roots | Decoction; Maceration | [2, 33] | Least concern | Native | NR | 2 (5.3%) | Muda (MA) [2], SO, MA, ZB, T, NA, CD, NI [33] |
| *Saccharum officinarum* L. | NR | Poaceae | Leaves | Decoction | [17] | NR | Introduced | - | 1 (2.6%) | Boane district (MP) [17] |
| *Salacia kraussii* Harv. | Bobo, bôbo, chibobo, chibobo, chipua, cuiche, cuixe, imbobo, imbono, m’pschicha, m’psincha, manpechincha, mantshisa, mapsincha, mápsincha, mapsisha, mapuija, matsincha, mbossi, mbossi, mpsisha, ngododo, p’sincha, pchicha, pchincha, pschicha, psicha, psincha, psincha, psinxa, psixa, tsyissa, tuiche | Celastraceae | Roots | Decoction | [3, 4] | NR | Native | NR | 2 (5.3%) | MP, GZ, IB [3] |
| *Schotia brachypetala* Sond. | NR | Fabaceae | NR | NR | [4] | Least concern | Native | NR | 1 (2.6%) | NR [4] |
| *Sclerocarya birrea* Hochst. | Canhi, canhu, ditsula, ncanhe, ncanhu, nkanhu, nkanyi, tsula, | Anacardiaceae | Leaves, roots, bark | Decoction (can include *Garcinia livingstonei* bark [16]. Scrape before decoction) | [4, 6-8, 10-12, 16, 26] | NR | Native | NR | 9 (23.7%) | Marracuene district (MP) [26], Inhaca Island (MP) [16], Matutuíne, MP [11], MP [12], MP and GZ [7], Chokwé and Bilene (GZ) [6], Massingir district (GZ) [8], Limpopo National Park (GZ) [10] |
| *Sclerocarya birrea* subsp. *caffra* (Sond.) Kokwaro (Synonym reported: *Sclerocarya caffra* Sond.) | Canhi, canho, cheteco, dangua, ditsula, ditsula, engongo, fula, impepo, linvula, mˊtula, m’fura, m’tula, marula, mecoco, medangwa, mefula, megongo, mepepe, mepepo, merula, metula, mfuura, mkoko, morula, motula, mtula, muchangua, mudangua, mudângua, muganu, mukoko, mutolo, mutonduoco, n’kanhy, n’koko, ncanhe, nfula, ngongo, nipepo, nkanhi, nkanye, ntsula, ocanheira, ocanheiro, ocanho, tchumbo, tsula, txiumbo, umpepo, unfula, unvula | Anacardiaceae | Roots | Decoction; Maceration; Add to porridge after grinding | [14, 23, 25] | NR | Native | NR | 3 (7.9%) | Magude district (MP) [14], MP [23], MP, GZ, IB, SO, MA, ZB, T, NA, CD, NI [25] |
| *Searsia dentata* (Thunb.) F.A.Barkley (Synonym reported: *Rhus dentata* Thunb.) | Bindaopinda, dambacerera, deiambeva, muteambeva | Anacardiaceae | Leaves, roots | Maceration | [2] | Least concern | Native | NR | 1 (2.6%) | Muda (MA) [2] |
| *Secamone punctulata* Decne. | Ximufane | Apocynaceae | Roots | Decoction | [5] | NR | NR | NR | 1 (2.6%) | Medicinal plant markets (MC) [5] |
| *Secamone schweinfurthii* K.Schum. (Synonym reported: *Secamone parvifolia* Bullock) | NR | Apocynaceae | NR | NR | [10] | NR | Native | NR | 1 (2.6%) | Limpopo National Park (GZ) [10] |
| *Securidaca longepedunculata* Fresen. (Synonym reported: *Securidaca longipedunculata*) | Mulhalhovo | Polygalaceae | Roots | NR | [35] | Least concern | Native | NR | 1 (2.6%) | Matutuíne district (MP) [35] |
| *Senegalia kraussiana* (Meisn. ex Benth.) Kyal. & Boatwr. (Synonym reported: *Acacia kraussiana* Meisn. ex Benth.) | Micaia, mungamazi | Fabaceae | Roots, stem bark | NR | [1] | NR | Native | NR | 1 (2.6%) | Katembe (MC) [1] |
| *Senna occidentalis* (L.) Link | Nhokane, nhokane tsongo, nhokane uculo, ndlha nhoka, ndlha nhokane | Fabaceae | Roots | Decoction | [5] | Least concern | Introduced | - | 1 (2.6%) | Medicinal plant markets (MC) [5] |
| *Senna petersiana* (Bolle) Lock | Demberembe, mudemberembe, nembe-nembe, | Fabaceae | Roots | Maceration | [10, 14, 34] | Least concern | Native | NR | 3 (7.9%) | Macate district (MA) [34], Boane and Magude (MP) [14], Limpopo National Park (GZ) [10] |
| *Senna* sp. | Mudemberembe, mudzepete, munhundunwa, | Fabaceae | Roots, leaves | Maceration | [2, 22] | * | * | * | 2 (5.3%) | Manica district (MA) [22], Muda (MA) [2] |
| *Sesamum eriocarpum* (Decne.) Byng & Christenh. (Synonym reported: *Dicerocaryum eriocarpum* (Decne.) Abels) | NR | Pedaliaceae | NR | NR | [10] | NR | NR | NR | 1 (2.6%) | Limpopo National Park (GZ) [10] |
| *Sonchus oleraceus* L. | Chinhahuasse, chinhamucaca, niacave | Asteraceae | Sap, roots | Maceration | [2] | NR | Introduced | - | 1 (2.6%) | Muda (MA) [2] |
| *Spirostachys africana* Sond. | Chilangamalho, libhandwa, mubhandwa, xilangamalho, xilate | Euphorbiacecae | Roots, stem | Decoction; Char the stem with chicken feces on charcoal and apply the resulting smoke to the anus | [1, 4-7] | Least concern | Native | NR | 5 (13.2%) | Medicinal plant markets (MC) [5], Katembe, MC [1], MP and GZ [7], Chokwé and Bilene (GZ) [6] |
| *Strophanthus kombe* Oliv. | Duruvane, kombe, kombi, machlamazaka, mulhabongue, sulo, unssulo, utsulo | Apocynaceae | Leaves | Decoction | [18] | NR | Native | NR | 1 (2.6%) | MP, GZ, IB, SO, MA, ZB, T, CD [18] |
| *Strychnos decussata* (Pappe) Gilg | Ntogetha, xinkwakwane, xinkwakwani | Loganiaceae | Roots | Decoction | [6, 7] | Least concern | Native | NR | 2 (5.3%) | MP and GZ [7], Chokwé and Bilene (GZ) [6] |
| *Strychnos henningsii* Gilg | Manono, xinderani | Loganiaceae | Roots (including root bark) | Decoction; Maceration | [1, 5, 6] | Least concern | Native | NR | 3 (7.9%) | Medicinal plant markets (MC) [5], Katembe, MC [1], Chokwé and Bilene (GZ) [6] |
| *Strychnos madagascariensis* Poir. | Inkwaka | Loganiaceae | Leaves, roots | Decoction (following grinding, combining with *Terminalia sericea* and *Vernonia colorata* leaves, then drying) | [19] | Least concern | Native | NR | 1 (2.6%) | Matutuíne district (MP) [19] |
| *Syzygium cordatum* Hochst. | Mulhu | Myrtaceae | Bark | Decoction | [16] | Least concern | Native | NR | 1 (2.6%) | Inhaca Island (MP) [16] |
| *Tabernaemontana elegans* Stapf | Cacho, cachuana, cachuane, cahlu, calho, calhu, calhuana, catchlo, catchlo-a-mutsui, cau-cau, chenga, dicassu, eracaraca, erracarraca, incasso, incassu, in-kasi-chonga, kahlu, kahlwana, kalhu, langodi, licachu, lilangode, limbo, m’cau-cau, macachuane, mcau-mcau, mcau-mcau, mucasso, muchene, mucshu, mukankaú, nangode, ncahlo, ncahlu, ncahlu, ncahu, ncalhu, nhama côbe, nhancaca, nkahlo, nkahlu, nkahlu nkashu, nyankaka, raca-raca, racaraca manalé moráre, racarraca, silangodi, umkhaslu, unkaasjo, unkhaslu, zangozango | Apocynaceae | Roots | Decoction; Infusion; Maceration;  Make porridge with maceration  (other plants can be added to decoction [16, 18]) | [5, 7, 12, 16, 18, 20, 21] | Least concern | Native | NR | 7 (18.4%) | Medicinal plant markets (MC) [5], Matutuíne (MP) [21], Inhaca Island (MP) [16], MP and GZ [7, 12, 20], MP, GZ, SO, MA, ZB, T, NA, CD, NI [18] |
| *Talinum caffrum* (Thunb.) Eckl. & Zeyh. | Magutlu | Talinaceae | Roots | Maceration | [6] | NR | Native | NR | 1 (2.6%) | Chokwé and Bilene (GZ) [6] |
| *Terminalia myrtifolia* (M.A.Lawson) Gere & Boatwr. | NR | Combretaceae | NR | NR | [10] | NR | Native | NR | 1 (2.6%) | Limpopo National Park (GZ) [10] |
| *Terminalia phanerophlebia* Engl. & Diels | Nkonola | Combretaceae | NR | NR | [12] | Least concern | Native | NR | 1 (2.6%) | MP [12] |
| *Terminalia sericea* Burch. ex DC. | Canzadzi, cassache, cassatgi, codoni, conola, cónola, conona, conono, cossange, cunona, gonôno, hai-hai, inconola, kassantje, kondla, kondla, konola, konono, m’susso, mecodome, mogonono, mpururu, muçaçai, mucoudône, mugodoni, muhai-hai, musasai, mussuco, mussuço, mussusso, mussussu, mususo, nalensi, niputua, nkonola, nkonolo, nkonono,nsunsu, sai-sai, sassai, sunsu | Combretaceae | Roots, leaves | Decoction (can also be used as enema [1]), infusion, maceration; make porridge with maceration [3]. Other plants can be added to decoction [1, 19, 24] including root of *Tabernaemontana elegans* [3]) | [1-8, 12, 16, 19, 24, 28, 32] | Least concern | Native | NR | 14 (36.8%) | Medicinal plant markets (MC) [5], Katembe (MC) [1], MC [24], Matutuíne district (MP) [19], Magude district (MP) [32], Inhaca Island (MP) [16], Marracuene and Boane, MP [28], MP and GZ [7] [12], Massingir district (GZ) [8], Chokwé and Bilene (GZ) [6], MP, GZ, IB, SO, MA, ZB, T, NA, CD, NI [3], Muda (MA) [2] |
| *Tetradenia riparia* (Hochst.) Codd (Synonym reported: *Iboza riparia* N.E.Br.) | Bozana | Lamiaceae | Roots, leaves | Decoction; Infusion | [28] | Least concern | Native | NR | 1 (2.6%) | Marracuene and Boane, MP [28] |
| *Thilachium africanum* Lour. | Chimuamarruca, chirica-cama, compfa, compha, kala nherere, m’sucamano, m’tatu, mahua, mamucapa,mathema, n odjesse, namecapa, namucapuano, nanelele, napucapa, nhendze, nyendza, nyendzi, taúa-nherére | Capparaceae | Roots | Decoction (can be mixed with roots of *Boscia salicifolia*) | [8, 20, 27] | Least concern | Native | NR | 3 (7.9%) | MP and GZ [20], Massingir district (GZ) [8], MP, GZ, IB, MA, ZB, T, NA, CD, NI [27] |
| *Tiliacora funifera* Oliv. | Chiwizila, Xiwizila | Menispermaceae | Roots | Decoction; Maceration | [5-7, 24] | NR | Native | NR | 4 (10.5%) | Medicinal plant markets (MC) [5], MC [24], MP and GZ [7], Chokwé and Bilene (GZ) [6] |
| *Trichilia emetica* Vahl | Kalhu, Kulhu | Meliaceae | Bark | Make enema after crushing, filtering, adding salt and ashes | [4, 11, 16] | Least concern | Native | NR | 3 (7.9%) | Inhaca Island (MP) [16], Matutuíne (MP) [11] |
| *Turraea nilotica* Kotschy & Peyr. | Mutangasua | Meliaceae | Roots | Maceration | [2] | Least concern | Native | NR | 1 (2.6%) | Muda (MA) [2] |
| *Vachellia karroo* (Hayne) Banfi & Galasso (Synonym reported: *Acacia karroo* Hayne) | Nkaya | Fabaceae | Stem | Maceration | [4, 6] | Least concern | Native | NR | 2 (5.3%) | Chokwé and Bilene (GZ) [6] |
| *Vachellia nilotica* (L.) P.J.H.Hurter & Mabb. (Synonym reported: *Acacia nilotica* (L.) Willd. ex Delile) | NR | Fabaceae | Roots | NR | [4, 20] | Least concern | Native† | NR | 2 (5.3%) | MP and GZ [20] |
| *Vangueria infausta* Burch. | Mapfilwa, maphilo, mphilwa | Rubiaceae | Roots | NR (only report is mixing with roots of other plants [16]) | [10, 12, 16, 32] | Least concern | Native† | NR | 4 (10.5%) | Inhaca Island (MP) [16], MP [12], Magude (MP) [32], Limpopo National Park (GZ) [10] |
| *Vernonia colorata* Drake | Inhatele, lile, mpalhacufa, navata, navate, navatha, nhatela, nhatella, nhatelo, nhatelu ya kwate, nhathela, nhathelo, pacha, pácha, pachacufa, pacha-kufa, pahlakufa, phacha, tsonzôro | Asteraceae | Roots, leaves | Decoction; Maceration (apart from ingestion, the maceration can also be applied to the anus). Mixtures may be added [1, 24] | [1, 6, 7, 14, 17, 19, 24, 33, 36, 37] | Least concern | Native† | NR | 10 (26.3%) | Katembe, MC [1], MC [24], Matutuíne district (MP) [19], Marracuene and Manhiça districts, MP [36], Boane district (MP) [14] [17], Chibuto District, GZ [37], Chokwé and Bilene (GZ) [6], MP and GZ [7], MP, GZ, SO, MA, ZB, T, NA, CD, NI [33] |
| *Volkameria glabra* (E.Mey.) Mabb. & Y.W.Yuan (Synonym reported: *Clerodendrum glabrum* E.Mey.) | Pechacuado, phembo, vumbanhe | Lamiaceae | NR | NR | [12] | Least concern | Native | NR | 1 (2.6%) | MP [12] |
| *Warburgia salutaris* (G.Bertol.) Chiov. | Xibaha | Canellaceae | Bark | Decoction; Infusion | [10, 28, 38] | Vulnerable↓ | Native | NR | 3 (7.9%) | Marracuene and Boane (MP) [28], Matutuíne and Namaacha (MP) [38], Limpopo National Park (GZ) [10] |
| *Ximenia americana* L. | Nthunduluka, thlathlangate wa mutsongo, tsenguele, tunduluca | Olacaceae | Roots | Decoction | [6, 12] | Least concern | Native† | NR | 2 (5.3%) | MP [12], Chokwé and Bilene (GZ) [6] |
| *Ximenia caffra* Sond. | NR | Olacaceae | NR | NR | [10] | Least concern↓ | Native† | NR | 1 (2.6%) | Limpopo National Park (GZ) [10] |
| *Xylotheca tettensis* (Klotzsch) Gilg | NR | Achariaceae | Roots, stem, leaves | NR | [15] | Least concern | Native† | NR | 1 (2.6%) | Ancuabe district (CD) [15] |
| *Zanthoxylum capense* Harv. | Manungwani | Rutaceae | Roots | Decoction | [6, 7] | Least concern | Native | NR | 2 (5.3%) | MP and GZ [7], Chokwé and Bilene (GZ) [6] |
| *Zanthoxylum humile* (E.A.Bruce) P.G.Waterman | NR | Rutaceae | NR | NR | [10] | NR | Native | NR | 1 (2.6%) | Limpopo National Park (GZ) [10] |
| *Zanthoxylum rhetsa* (Roxb.) DC. | NR | Rutaceae | Roots, stem, leaves | NR | [15] | Least concern | NR | NR | 1 (2.6%) | Ancuabe district (CD) [15] |
| *Zanthoxylum* sp. | Munungwana | Rutaceae | NR | NR | [12] | * | * | * | 1 (2.6%) | MP [12] |
| *Ziziphus mucronata* Willd. | M’phafa, Mphafa | Rhamnaceae | Roots | Decoction | [4, 6, 7] | Least concern | Native | NR | 3 (7.9%) | MP and GZ [7], Chokwé and Bilene (GZ) [6] |
| Unidentified | Chicodoro | - | Roots | Decoction | [2] | * | * | * | 1 (2.6%) | Muda (MA) [2] |
| Unidentified | Chifiti | - | Leaves | Decoction | [34] | * | * | * | 1 (2.6%) | Macate district (MA) [34] |
| Unidentified | Chimbambara | - | Bark, roots | Maceration | [2] | * | * | * | 1 (2.6%) | Muda (MA) [2] |
| Unidentified | Chinhahuaje | - | Roots | Decoction | [2] | * | * | * | 1 (2.6%) | Muda (MA) [2] |
| Unidentified | Churuquira | - | Roots | Decoction | [2] | * | * | * | 1 (2.6%) | Muda (MA) [2] |
| Unidentified | Magazine | - | Roots | Decoction | [5] | * | * | * | 1 (2.6%) | Medicinal plant markets (MC) [5] |
| Unidentified | Mungurahue | - | Roots | Decoction | [2] | * | * | * | 1 (2.6%) | Muda (MA) [2] |
| Unidentified | Nhacutslwani | - | Roots | Decoction | [5] | * | * | * | 1 (2.6%) | Medicinal plant markets (MC) [5] |
| Unidentified | Towane | - | Roots | Decoction | [5] | * | * | * | 1 (2.6%) | Medicinal plant markets (MC) [5] |
| Unidentified | Xicotela | - | NR | NR | [32] | * | * | * | 1 (2.6%) | Magude district (MP) [32] |

**NR** – Not reported, **IB** – Inhambane, **MP** – Maputo Province, **GZ** – Gaza, **MA** – Manica, **CD** – Cabo Delgado, **MC** – Maputo City, **NA** – Nampula, **NI** – Niassa, **SO** – Sofala, **T** – Tete, **ZB** – Zambezia

* There is no information because the plant was not identified to species level

† Selected varieties or subspecies. Among those listed as near endemic, the following varieties were identified: *Acridocarpus natalitius* A.Juss. var. *linearifolius*, *Dichrostachys cinerea* (L.) Wight & Arn. var. *pubescens* and *Ozoroa obovata* (Oliv.) R.Fern. & A.Fern. var. *elliptica*

‡ Conservation status not separately assessed; refers to the conservation status of the species as listed in the IUCN Red List as least concern

↓ Decreasing population

**References**

1. Dai ML: **Estudo dos padrões de plantas Medicinais na localidade de Catembe**. Universidade Eduardo Mondlane; 1997.

2. Bruschi P, Morganti M, Mancini M, Signorini MA: **Traditional healers and laypeople: a qualitative and quantitative approach to local knowledge on medicinal plants in Muda (Mozambique)**. *Journal of Ethnopharmacology* 2011, **138**(2):543-563.

3. Jansen PCM, Mendes O: **Plantas Medicinais: seu uso tradicional em Moçambique [Medicinal plants: Their traditional use in Mozambique] (Tomo 4)**: Gabinete de Estudos de Medicina Tradicional.; 1991.

4. Bandeira SO, Gaspar F, Pagula FP: **African ethnobotany and healthcare: emphasis on mozambique**. *Pharm Biol* 2001, **39 Suppl 1**:70-73.

5. Barbosa F, Hlashwayo D, Sevastyanov V, Chichava V, Mataveia A, Boane E, Cala A: **Medicinal plants sold for treatment of bacterial and parasitic diseases in humans in Maputo city markets, Mozambique**. *BMC Complementary Medicine and Therapies* 2020, **20**(1):19.

6. Maueia CG: **Estudo etnobotânico das plantas medicinais utilizadas no tratamento da malária, tuberculose e amebiase na província de Gaza**. Maputo: Universidade Eduardo Mondlane; 2007.

7. Santiago EM: **Estudo Fitoquímico das plantas medicinais usadas no tratamento de doenças causadas por protozoários e microbactérias. Avaliação dos alcalóides de grupo de berberina nas plantas medicinais**. Maputo: Universidade Eduardo Mondlane; 2007.

8. Ribeiro A, Romeiras MM, Tavares J, Faria MT: **Ethnobotanical survey in Canhane village, district of Massingir, Mozambique: medicinal plants and traditional knowledge**. *Journal of Ethnobiology and Ethnomedicine* 2010, **6**:33.

9. Matavele J, Habib M: **Ethnobotany in Cabo Delgado, Mozambique: Use of Medicinal Plants**. *Environment, Development and Sustainability* 2000, **2**(3):227-234.

10. Nicosia E, Valenti R, Guillet A, Mondlane TDSM, Malatesta L, Odorico D, Tallone G, Attorre F: **An ethnobotanical survey in the Limpopo National Park, Gaza province, Mozambique: traditional knowledge related to plant use**. *Rendiconti Lincei Scienze Fisiche e Naturali* 2022, **33**(2):303-318.

11. Senkoro AM, Barbosa FMA, Moiane SF, Albano G, de Barros AIR: **Bark Stripping from Forest Tree Species in Madjadjane, Southern Mozambique: Medicinal Uses and Implications for Conservation**. *Natural Resources* 2014, **5**(5):192-199.

12. Fumane J, Candido A, Barbosa F, Boana C, Dungo J, Mateus R, Bandeira B, Acha V, Gaspar F: **Pesquisa Etnobotânica de Plantas Medicinais Utilizadas pelos Praticantes da Medicina Tradicional no tratamento de doenças associadas ao HIV/SIDA**. In*.*: FDC; 2003.

13. Novela JA: **Plantas Medicinais: Seus usos e estado de conservação na Aldeia de Chibotana parque Nacional de Limpopo Distrito de Massingir, Gaza**. Maputo: Universidade Eduardo Mondlane; 2006.

14. Gaspar F: **Etnobotânica e percepção cultural, em relação ao cultivo de plantas medicinais, na Província de Maputo**. Maputo: Universidade Eduardo Mondlane; 2000.

15. Muchaia AJ, Nanvonamuquitxo SJA: **Levantamento etnobotânico de plantas medicinais utilizadas pela comunidade de Nacuale, no parque nacional das Quirimbas, Moçambique**. *Nativa* 2021, **9**(5):605-611.

16. Barbosa FMAB: **Uma avaliação do valor das árvores para a população da Ilha da Inhaca**. Maputo: Universidade Eduardo Mondlane; 1995.

17. Manuel MF, Lobo F: **Levantamento de Plantas Medicinais usadas na localidade de Mahubo, Município de Boane, Província de Maputo**. *Revista Moçambicana de Etnociências-Mukobwa* 2019, **1**:5-17.

18. Jansen PCM, Mendes O: **Plantas Medicinais: seu uso tradicional em Moçambique [Medicinal plants: Their traditional use in Mozambique] (Tomo 2)**: Gabinete de Estudos de Medicina Tradicional; 1983b.

19. Sousa IM: **Plantas medicinais da localidade de Catembe, sua caracterização e aspectos sobre a sua comercialização**. Maputo: Universidade Eduardo Mondlane; 2007.

20. Mulhovo SF: **Plantas medicinais utilizadas no tratamento de asma em Moçambique.** Maputo: [Instituto Superior Pedagógico; 1994.

21. BIOFUND: **Relatório sobre a disponibilidade, ecologia e sistemas de uso actual de plantas indígenas de Matutuine**. In*.* <https://biblioteca.biofund.org.mz/wp-content/uploads/2019/01/1548415090-E501_Relat%C3%B3rio.pdf>.

22. Senkoro AM: **Impacto da exploração do ouro na macroflora no Monte Munhena e no Vale Revué, Distrito de Manica**. Maputo: Universidade Eduardo Mondlane; 2001.

23. Verzar R, Petri G: **Medicinal plants in Mozambique and their popular use**. *J Ethnopharmacol* 1987, **19**(1):67-80.

24. Jonasse C: **Usos de fitoterápicos no tratamento de crianças de até 5 anos no Hospital Central de Maputo**. Eduardo Mondlane University; 2013.

25. Jansen PCM, Mendes O: **Plantas Medicinais: seu uso tradicional em Moçambique [Medicinal plants: Their traditional use in Mozambique] (Tomo 1)**: Gabinete de Estudos de Medicina Tradicional; 1983a.

26. Manjate M: **Um estudo da diversidade botânica e padrões de uso das plantas de Chificundzi**. Maputo: Universidade Eduardo Mondlane; 1996.

27. Jansen PCM, Mendes O: **Plantas Medicinais: seu uso tradicional em Moçambique [Medicinal plants: Their traditional use in Mozambique] (Tomo 3)**: Gabinete de Estudos de Medicina Tradicional; 1990.

28. Chilemba N: **Algumas plantas medicinais e tóxicas Identificadas no Hospital Central de Maputo e Hospital Geral José Macamo**. Universidade Eduardo Mondlane; 1997.

29. Cande SEP: **Plantas medicinais usadas para tratamento de "Nyocane" no Distrito de Homoine-Província de Inhambane**. Maputo: Universidade Eduardo Mondlane; 2006.

30. Halafo JS: **Estudo da planta *Warburgia salutaris* (Fertol. F.) Chiov. na floresta Licuáti. estado de conservação e utilização pelas comunidades locais**. Maputo: Universidade Eduardo Mondlane; 1996.

31. Conde P, Figueira R, Saraiva S, Catarino L, Romeiras M, Duarte MC: **The Botanic Mission to Mozambique (1942-1948): contributions to the knowledge of the medicinal flora of Mozambique**. *História, Ciências, Saúde-Manguinhos* 2014, **21**(2):1-47.

32. Simone M: **Estudo de plantas medicinais em uso pelas comunidades locais no ponto administrativo de Mahel e sua propagação**. Maputo: Universidade Eduardo Mondlane; 2001.

33. Jansen PCM, Mendes O, Da Silva MC: **Plantas Medicinais: seu uso tradicional em Moçambique [Medicinal plants: Their traditional use in Mozambique] (Tomo 5)**: Gabinete de Estudos de Medicina Tradicional.; 2001.

34. Aparicio H, Hedberg I, Bandeira S, Ghorbani A: **Ethnobotanical study of medicinal and edible plants used in Nhamacoa area, Manica province–Mozambique**. *South African Journal of Botany* 2021, **139**:318-328.

35. Nuvunga RS: **Estudo da planta *Securidaca longipedunculata* Fresen em Santaca: Estado de conservação e uso pelas comunidades locais**. Maputo: Universidade Eduardo Mondlane; 1998.

36. Casmo V: **Diversidade, Mapeamento e Utilidade das Plantas da Bacia do Rio Incomati**. Maputo: Universidade Eduardo Mondlane; 2004.

37. Manjate AOM: **Avaliação ecológica do estado de conservação da vegetação das terras húmidas de Missavene Distrito de Chibuto, Provincia de Gaza**. Maputo: Universidade Eduardo Mondlane; 2009.

38. Senkoro AM, Shackleton CM, Voeks RA, Ribeiro AI: **Uses, Knowledge, and Management of the Threatened Pepper-Bark Tree (Warburgia salutaris) in Southern Mozambique**. *Economic Botany* 2019, **73**(3):304-324.
